# Arthroscopic treatment of septic arthritis of the elbow in a 5-year-old boy: A case report

**DOI:** 10.1016/j.ijscr.2025.111041

**Published:** 2025-02-12

**Authors:** Fengyong Mao, Lei Ni, Li Ju

**Affiliations:** Department of Orthopaedics Surgery, Children's Hospital of Nanjing Medical University, Nanjing 210000, Jiangsu Province, China

**Keywords:** Septic arthritis, Pediatric, Elbow, Case report, Arthroscopy

## Abstract

**Introduction:**

Septic arthritis of the elbow in children is rare but can lead to permanent joint damage if not treated promptly.

**Case presentation:**

We report a case of a 5-year-old boy diagnosed with septic arthritis of the elbow, treated with arthroscopy using a 4.5-mm arthroscope, followed by antibiotic therapy. Pre- and post-operative pain and functional outcomes were evaluated using the Visual Analog Scale (VAS) and the Mayo Elbow Performance Score (MEPS).

**Clinical discussion:**

The patient achieved full range of motion within two weeks and completes recovery at six months.

**Conclusion:**

This case demonstrates that arthroscopy is an effective, minimally invasive option for managing pediatric elbow septic arthritis.

## Highlights

• Septic arthritis of the elbow in children is particularly uncommon but can lead to permanent joint damage if untreated.
• Conventional 4.5 mm arthroscopic instruments used, not smaller hip/elbow scopes.
• This case demonstrates arthroscopy as an effective, minimally invasive option for managing pediatric elbow septic arthritis.

## 1 Introduction

Septic arthritis in children is a relatively uncommon yet serious condition that can result in permanent joint damage if not diagnosed and managed promptly. Although septic arthritis most commonly affects the hip, knee, and ankle joints, elbow involvement is less frequent [1]. Conventional management typically includes joint aspiration and open arthrotomy [2]. However, recent studies have highlighted the benefits of arthroscopic surgery for septic arthritis in both adults and pediatric patients across various joints [3]. Despite these advancements, the use of arthroscopy for septic arthritis of the elbow in children has not been widely reported. This case report describes the successful management of septic arthritis in a 5-year-old Chinese male of Han ethnicity through arthroscopic debridement, demonstrating its effectiveness in treating pediatric elbow joint infections.

## 2 Case presentation

A 5-year-old Chinese male of Han ethnicity presented to an outside hospital with a 7-day history of right elbow pain. The initial diagnosis was elbow trauma, although there was no clear history of trauma or fever. Despite conservative treatment with rest and ice, the symptoms persisted, prompting a second visit to our hospital. The patient was afebrile, with no systemic symptoms or significant past medical history, including immune disorders or prior surgeries. Family history was unremarkable, with no hereditary or systemic conditions.

**Fig. 1:**
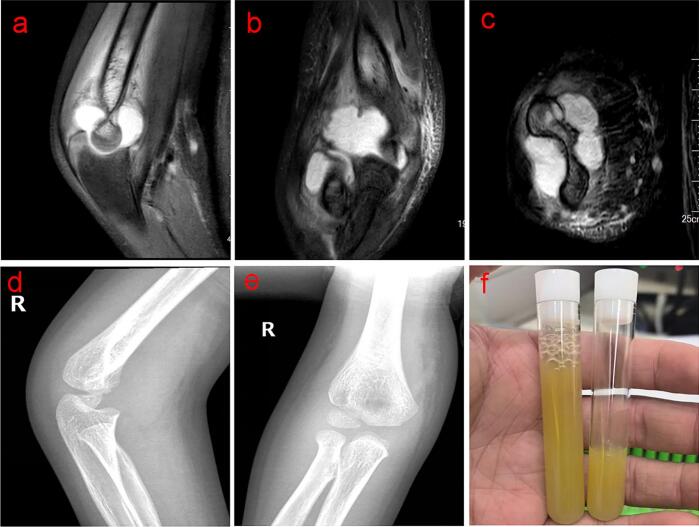
The preoperative status of the patient's elbow joint. a, b, c: MRI demonstrates joint effusion, synovial thickening and swelling; d, e: plain imaging shows no significant abnormalities; f: joint aspirated the cloudy synovial fluid.

Physical examination revealed tenderness and swelling around the right elbow, with a limited range of motion from 30° to 90°. The patient reported a pain level of 8/10 on the Visual Analog Scale (VAS) and had a Mayo Elbow Performance Score (MEPS) of 45. Laboratory tests showed elevated inflammatory markers, including a white blood cell count of 11.49 × 10^9/L, a C-reactive protein (CRP) level of 34.83 mg/L and an erythrocyte sedimentation rate (ESR) level of 79 mm/h. Magnetic resonance imaging (MRI) of the right elbow revealed joint effusion and synovial thickening (Fig. 1a, b, c). Plain radiographs showed soft tissue swelling without evidence of fracture or osteomyelitis (Fig. 1d, e). Joint aspiration yielded slightly cloudy synovial fluid (Fig. 1f), and cytopathology revealed numerous inflammatory cells on the smear, with neutrophils comprising 70 %, lymphocytes 10 %, and monocytes 20 %. Given these findings, a diagnosis of septic arthritis of the right elbow was made, and the patient was admitted for further management.

## 3 Diagnosis and management

**Fig. 2:**
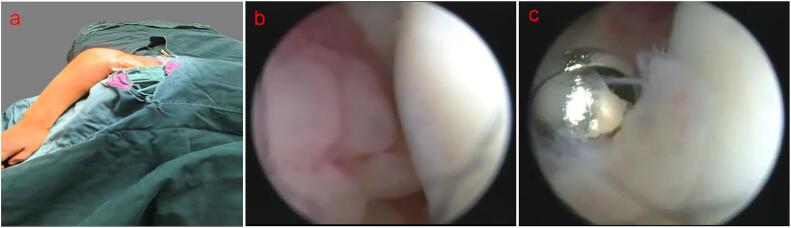
Intraoperative environments. a: The patient in the lateral decubitus position; b: the joint cavity can be achieved seamlessly via this approach, synovial proliferation was significantly noted; c: the synovium removed by shaver.

Given the persistently elevated inflammatory markers and the patient's lack of improvement with conservative measures, surgical intervention was indicated on the third day of hospitalization. The patient underwent arthroscopic debridement of the right elbow joint using a 4.5-mm, 30° arthroscopic system (Smith & Nephew), originally designed for knee joints. The procedure was performed with the patient in the lateral decubitus position (Fig. 2a). A posterior portal was created 1 cm above the olecranon process of the ulna for joint assessment, and a posterolateral portal was established using the outside-in technique under direct visualization. Significant synovial proliferation was noted within the joint during arthroscopic visualization, but only the minimal amount of synovium obstructing the view was removed (Fig. 2b). Debridement was carried out under continuous irrigation with 2000 mL of saline. Post-operatively, the elbow was immobilized with a splint to protect the joint. The VAS score was 5 on the first day after surgery.

Post-operative cultures from the initial joint aspiration identified *Streptococcus pyogenes*, prompting the continuation of intravenous cefazolin therapy. One week post-operatively, CRP levels returned to normal, and ESR was 25 mm/h. Intravenous antibiotics were discontinued, and oral antibiotics were initiated. The splint was also removed at this time and pain was rated 2 on the VAS. The patient showed significant clinical improvement post-surgery, with reduced swelling and pain, and regained full range of motion within 2 weeks. MEPS improved to 100 at the 6-month follow-up and no pain was recorded.

The reporting of this study adheres to the SCARE criteria for surgical case reports, ensuring the transparency and quality of the case report. We have meticulously documented the presentation of the case, the operative procedure, the outcomes, and subsequent follow-up in accordance with the latest SCARE 2023 guidelines [4].

## 4 Follow-up and outcome

**Fig. 3:**
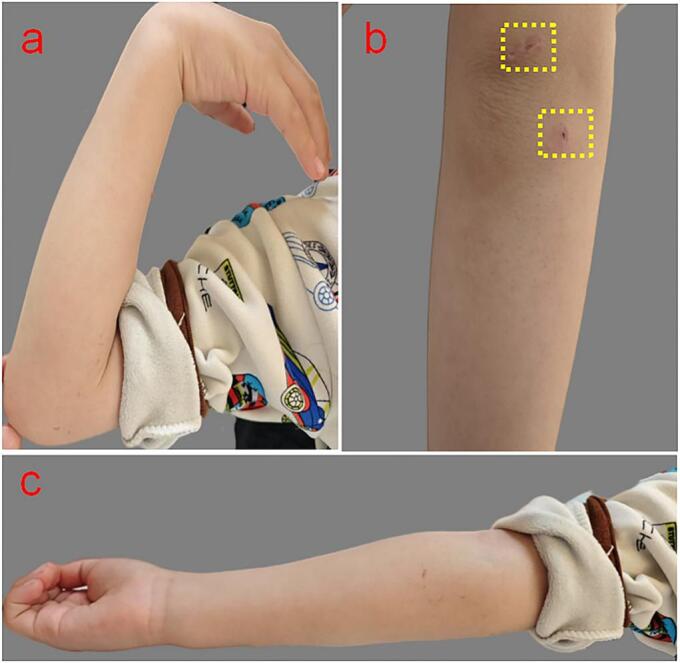
The outcome of the patient's elbow joint. a, b, c: No signs of joint stiffness, no limitations of the joint flexion and extension activities and outstanding arthroscopic incisions (the yellow dashed box). (For interpretation of the references to colour in this figure legend, the reader is referred to the web version of this article.)

At the 6-month follow-up, the patient demonstrated full functional recovery of the right elbow, with no signs of osteoarthritis, joint stiffness, or pain. The arthroscopic incisions were healing well (Fig. 3a, b, c).

## 5 Discussion

In pediatric septic arthritis, early diagnosis and prompt drainage are critical to prevent joint destruction and permanent disability [5]. While joint aspiration is often the initial diagnostic step, it may not always be sufficient to fully eradicate infection in more severe cases [6]. Open arthrotomy has long been advocated as the standard surgical treatment for septic arthritis. With arthrotomy, a clearer view of the joint is obtained, and sufficient irrigation can be performed. However, it may lead to prolonged recovery times, a higher risk of avascular necrosis, and associated complications, such as joint stiffness [2]. Recent studies suggest that arthroscopic debridement could be a more effective treatment option, especially when early intervention is crucial [7,8]. Compared to open arthrotomy, arthroscopy offers several advantages, including minimal invasiveness, reduced postoperative pain, and faster recovery times [9]. This aligns with our patient's rapid recovery, as full range of motion was regained within two weeks after surgery. However, despite these positive outcomes, the limitations of arthroscopy in pediatric septic arthritis, particularly in smaller joints, are associated with technical challenges and equipment constraints [2]. Incomplete debridement and the risk of chondral damage remain potential risks of arthroscopy, although these issues were not observed in our case [10].

In our case, we selected the lateral decubitus position for the surgical procedure to ensure optimal exposure of the elbow joint while facilitating easier access to the patient's airway and improving control during anesthesia. Pediatric elbows are significantly smaller than those of adults, presenting unique challenges in arthroscopic surgery. A recent report described the use of a wrist arthroscope for treating elbow infection in a 4-year-old child, emphasizing the need for specialized equipment and techniques when operating on small joints [11]. However, the wrist arthroscope is not available in all medical institutions, limiting its broader application. In our case, we used a 4.5 mm arthroscope, traditionally employed for knee arthroscopy, demonstrating that even a knee scope can be effectively applied in pediatric elbow joint procedures. The first portal was created approximately 1 cm above the olecranon process, and the second portal was established under direct visualization between the lateral epicondyle and the radial head, providing adequate joint visualization and sufficient operating space. A 3.5 mm shaver was used to remove only the synovium obstructing the view, ensuring minimal post-operative bleeding.

This case demonstrates the successful use of arthroscopic surgery for septic arthritis of the elbow in a pediatric patient. The primary conclusion is that arthroscopy has potential as a first-line treatment for pediatric septic arthritis, particularly in small joints like the elbow. However, the primary limitation of this report is its focus on a single patient. Further studies with larger sample sizes are necessary to fully establish the safety and efficacy of arthroscopic intervention for pediatric elbow septic arthritis. Additionally, long-term follow-up studies are crucial to evaluate potential long-term complications, such as the development of osteoarthritis.

## 6 Conclusion

This case demonstrates the successful use of arthroscopic surgery to treat pediatric septic arthritis of the elbow, resulting in favorable clinical outcomes. Arthroscopic debridement is a minimally invasive and effective treatment for joint infections, particularly in small joints like the elbow. Larger clinical trials are needed to validate these findings and assess its broader application in pediatric orthopaedics.

## Author contribution

**Fengyong Mao**: Writing- original draft, Writing – review & editing. **Lei Ni**: Conceptualization, Supervision. **Li Ju**: Conceptualization, Supervision, Writing – review &editing.

## Consent

Written informed consent was obtained from the patient's legal guardian for publication of this case report and any accompanying images.

## Ethical approval

The study was approved by the ethics committee of the Children's Hospital of Nanjing Medical University (202401017-1).

## Guarantor

The Guarantor is Ju Li.

## Research registration number

Our study is not ‘First in Man’ studies and does not meet the registration criteria for Research Registry.

## Funding

N/A.

## Conflict of interest statement

All authors declare that there is no conflict of interests exists.

## Data Availability

Available from corresponding author on reasonable request.
